# Complete Genome Sequence of *Bos taurus* Papillomavirus Type 1, Isolated in Morocco

**DOI:** 10.1128/genomeA.00979-17

**Published:** 2017-09-21

**Authors:** Guy L. Regnard, Asanda Matiso, Latif Mounir, Tarik Embarki, Inga I. Hitzeroth, Edward P. Rybicki

**Affiliations:** a Biopharming Research Unit, Department of Molecular and Cell Biology, University of Cape Town, Rondebosch, South Africa; b MCI Santé Animale, ZI Sud-Ouest, Mohammédia, Morocco; c Institute of Infectious Disease and Molecular Medicine, Faculty of Health Sciences, University of Cape Town, Observatory, South Africa

## Abstract

*Bos taurus* papillomaviruses infect cattle, which has both animal health and economic consequences. This is the first report and sequence of *Bos taurus* papillomavirus type 1 isolated from warts in cattle in Morocco. The double-stranded DNA (dsDNA) genome was 7,945 bp in size, and eight open reading frames were identified.

## GENOME ANNOUNCEMENT

*Bos taurus* (formerly bovine) papillomaviruses (BPV) belong to the family *Papillomaviridae*, and to date, 21 distinct BPV types have been identified (1, 2). Of these, BPV-1, BPV-2, and BPV-13 belong to the genus *Deltapapillomavirus* (3). The first full-length genome of BPV-1 was described in 1982 and is the type reference genome at the *Papillomavirus* Episteme site (pave.niaid.nih.gov) (4). The genome is a circular double-stranded DNA (dsDNA) molecule that is divided into an early (E) region with six open reading frames (ORFs), a late (L) region with two ORFs, and a noncoding region (NCR) (2). Cattle infected with BPV-1 are at risk of cancer and papillomatosis in the form of multiple benign epithelial proliferative lesions (5). In addition to the health implications, infection with BPV-1 can have economic consequences, especially for the dairy industry (5, 6).

Toward the end of 2015, sporadic cases of epithelial lesions affecting cattle on farms around Ain Harrouda, Morocco, were reported. Five to 10 cattle used for dairy and meat were affected. In January 2016, a lesion (50 mm in diameter) was completely excised with local treatment from a diagnosed adult crossbred cow (piebald) and stored (−80°C).

Total DNA was extracted from the lesion using a DNeasy blood and tissue kit (Qiagen, Germany) and prepared per the manufacturer’s instructions. Two overlapping 4.1-kb sections of the genome were amplified by PCR using Phusion high-fidelity DNA polymerase (New England BioLabs, USA) and the primer sets 5′-GGTTGGACTGTCTGTGGTG-3′ and 5′-AGGTCATAGGCACTGGCAC-3′ and 5′-CCCTGCTCAGATTTTATATGG-3′ and 5′-GAAAGTCTTTGACCATGCAC-3′, based on the sequence with GenBank accession number AB626705. The thermocycling conditions were initial denaturation at 98°C for 30 s, 30 cycles of 10 s at 98°C, 30 s at 55°C, and 150 s at 72°C, and final elongation at 72°C for 5 min. The amplified DNA was cloned using the CloneJET PCR cloning kit (Thermo Fisher Scientific, USA) per the manufacturer’s instructions, and the sequence of the DNA was determined using primer walking (Macrogen, South Korea). The sequences were assembled using DNAman version 5.2.9 (Lynnon BioSoft).

The full-length genome was compared to the reference BPV-1 genome (GenBank accession number X02346). The sequence was identical in size to the reference genome at 7,945 bp, and eight open reading frames were identified, containing the early genes E1, E2, E4, E5, E6, and E7 and the late genes L1 and L2. The sequence differed from the reference genome by a total of 36 nucleotides, with nonsynonymous mutations affecting the E1, E2, L1, and L2 genes. One insertion affected both the E2 and E4 genes, and there were two deletions and one insertion within the NCR. Our sequence shared greater than 99.4% identity with the reference sequence and 99% identity with other complete BPV-1 genome sequences in GenBank.

This is the first report of a full-length genome of any BPV from Morocco. The report indicates the highly conserved nature of BPV-1 in comparison to the reference sequence described decades earlier and on another continent.

### Accession number(s).

The complete genome sequence for *Bos taurus* papillomavirus type 1 was deposited in GenBank under the accession number KY746722.
